# Collinearity analysis of *Brassica* A and C genomes based on an updated inferred unigene order

**DOI:** 10.1016/j.dib.2015.01.004

**Published:** 2015-02-10

**Authors:** Ian Bancroft, Fiona Fraser, Colin Morgan, Martin Trick

**Affiliations:** John Innes Centre, Norwich Research Park, Norwich NR4 7UH, UK

## Abstract

This data article includes SNP scoring across lines of the *Brassica napus* TNDH population based on Illumina sequencing of mRNA, expanded to 75 lines. The 21, 323 mapped markers defined 887 recombination bins, representing an updated genetic linkage map for the species. Based on this new map, 5 genome sequence scaffolds were split and the order and orientation of scaffolds updated to establish a new pseudomolecule specification. The order of unigenes and SNP array probes within these pseudomolecules was determined. Unigenes were assessed for sequence similarity to the A and C genomes. The 57, 246 that mapped to both enabled the collinearity of the A and C genomes to be illustrated graphically. Although the great majority was in collinear positions, some were not. Analyses of 60 such instances are presented, suggesting that the breakdown in collinearity was largely due to either the absence of the homoeologue on one genome (resulting in sequence match to a paralogue) or multiple similar sequences being present. The mRNAseq datasets for the TNDH lines are available from the SRA repository (ERA283648); the remaining datasets are supplied with this article.

**Table:** Specifications table

Subject area	Biology

More specific subject area	*Plant genome organisation*
Type of data	*Tables (in the form of MS Excel spreadsheets) providing SNP marker scoring strings, genetic linkage map pseudomolecule specification based on genome sequence scaffolds, anchoring positions in the pseudomolecules for unigens and SNP array probes, and a figure illustrating the collinearity of the A and C genomes*.
How data was acquired	*mRNAseq data were derived by sequencing on Illumina HiSeq2000 platform*
Data format	*The data accompanying this article are provided in MS Excel spreadsheets*
Experimental factors	*Seed of the Doubled Haploid (DH) lines were sown into Scotts Levington F1 compost and grown on under long day glass house conditions. Plants were pricked out after 11 days into Levington M2 compost. Leaves were harvested 15 days after pricking out, 21 days after sowing. Leaf harvest was carried out as close to the midpoint of the light period as possible. The first true leaf of each plant was excised as close to the petiole as possible. Leaf samples were pooled and frozen in liquid nitrogen, giving a final harvest of four pooled leaf samples per DH line, from which RNA was purified*.
Experimental features	*SNP markers were scored using leaf mRNAseq data from an expanded Doubled Haploid mapping population and used for linkage map construction. The map was then used to improve pseudomolecules representing genome organisation inB. napus*, *which were then used for integration of sequence-based resources and analysis of genome collineraity*.
Data source location	n/a
Data accessibility	*SRA accession ERA283648*http://www.ncbi.nlm.nih.gov/sra/?term=ERA283648

Value of the data

• Provides further mRNAseq data, increasing the depth of transcriptome sequence available.
• Provides an updated genetic linkage map for *B. napus.*
• Provides an updates pseudomolecule resource to represent genome organisation in *B. napus.*
• Provides insights into the collinearity of the *Brassica* A and C genomes.
• Provides insights into the reasons for apparent gene-level breakdown of genome collinearity as assessed using sequence similarity searches.

## 1 Experimental design, materials and methods

Genome-wide association studies (GWAS) focus on panels of genetically diverse lines and exploit historical recombination between loci. Where recombination between loci is observed rarely, those loci are said to be in Linkage Disequilibrium (LD). The approach of identifying markers in LD with loci controlling traits is an important tool in human genetics studies and has been applied successfully in plants with well-established genome resources: maize, rice and *Arabidopsis thaliana* [1–5]. In crop science, GWAS can be used to understand the precise molecular basis of trait variation by identifying causative mutations, or at least to provide tightly linked markers to aid marker-assisted selection. To overcome the problem of lack of genome sequence resources, mRNAseq-based approaches have been developed for the polyploid crop species *Brassica napus* that enable high throughput identification of single nucleotide polymorphism (SNP) markers [6] and high density linkage map construction [7]. The linkage map enabled the development of a genomic framework; comprising genome sequence scaffolds from the progenitor species of *B. napus* (*Brassica rapa* and *Brassica oleracea*) rearranged into “pseudomolecules” representative of genome organisation of *B. napus*, which were then used successfully to underpin GWAS studies [8].

The linkage map underpinning the *B. napus* pseudomolecule arrangement was based on a mapping population of only 37 genotyped lines. This limited the resolution of the resource as the 21, 323 mapped markers defined only 527 recombination bins [7]. By increasing the size of the genotyped population to 75 lines, we aimed to increase the resolution of the linkage map, enabling improvement of the pseudomolecule resource (to version 4) by improving the determination of genome sequence scaffold order and orientation, and by detecting and splitting additional chimeric assemblies.

To increase the resolution of the genetic linkage map of *B. napus* based on SNP markers scored in unigene sequences, we grew 38 additional lines of the TNDH population: seed of the Doubled Haploid (DH) lines was sown into Plantpak 9 cm pots containing Scotts Levington F1 compost (Scotts, Ipswich, UK) and covered with a plastic propagator lid. The seeds were germinated and grown on under long day glass house conditions (16 h photoperiod) at 15 °C (400W HQI metal halide lamps). Plants were pricked out after 11 days into Plantpak P15 modules containing Scotts Levington M2 compost and arranged into a four block, one way randomized design with one plant of each of the DH lines per block and randomized within each block. Leaves were harvested 15 days after pricking out, 21 days after sowing. Leaf harvest was carried out as close to the midpoint of the light period as possible. The first true leaf of each plant was excised as close to the petiole as possible and the weight was recorded. Leaf samples for each Brassica line from each experimental block were pooled and frozen in liquid nitrogen, giving a final harvest of four pooled leaf samples per DH line.

From these lines, we purified RNA: RNA was prepared by grinding tissue in liquid nitrogen and extracting the RNA using the E.Z.N.A. Plant RNA Kit (Omega Bio-Tek Inc.) according to the manufacturer׳s protocol. RNA concentration was measured using  μl of each RNA sample on the NanoDrop ND-1000 Spectrophotometer. RNA quality was assessed by running  μl of each RNA sample on an Agilent RNA 6000 Nano LabChip (Agilent Technology 2100 Bioanalyzer), samples with an RNA Integrity Number (RIN) value greater than 8 were deemed acceptable according to the Illumina mRNA-Seq protocol.

From the purified RNA Illumina mRNAseq data was produced by The Genome Analysis Centre, Norwich on the Illumina HiSeq platform, with 100-base single end reads. The sequence reads were, trimmed to 80 bases and down-sampled to 28M reads per sample (to retain comparability with the earlier data), aligned to unigenes and scored for SNPs as described previously for an initial panel of 37 TNDH lines [7]. The scoring strings were used to extend those produced previously for 21, 323 SNP markers scored across the 37 TNDH lines used originally, and produce an updated linkage map. The data were used to refine the order of markers (Additional file 1 for the A genome, Additional file 2 for the C genome), increasing the number of recombination bins identified from 527 defined by the original 37 lines [7] to 887 defined by the full 75 lines. Consensus marker strings were defined for each of these recombination bins and a revised linkage map was constructed (Additional file 3).

Analysis of best sequence similarity matches in the *B. rapa* and *B. oleracea* genome sequence scaffolds of the unigenes in which SNP scored indicated that 5 of those scaffolds were chimeric (i.e. markers scored either side of position representing a breakdown in collinearity with the *A. thaliana* genome mapped to different places in the genome) so could be split (Additional file 4). The order and orientation of further genome sequence scaffolds could also be confirmed, enabling the updating of the specification of scaffold order and orientation (Additional file 5) to establish an updated (version 4) pseudomolecule resource comprising 736,647,178 bp of sequence data. These were then used to infer the order of unigenes and position the probe flanking sequences for the *Brassica* research community׳s Brassica 50K Illumina^®^ Infinium SNP array, thus integrating these two genotyping resources. The result is an inferred order of 86,429 sequences (61,757 unigenes; 24,672 array probes) representing potential markers in the A genome (Additional file 6) and 87,426 sequences (64,293 unigenes; 23,133 array probes) representing potential markers in the C genome (Additional file 7).

**Fig. 1:**
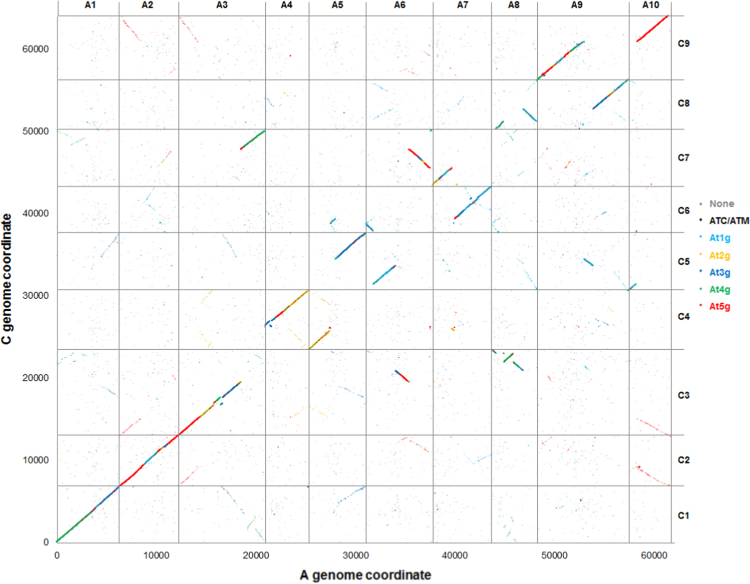
Collinearity of *B. napus* A and C genomes. The positions of best sequence matches in the A and C genome pseudomolecules are plotted for 57,246 unigenes that can be positioned (threshold value 1E−30) in both. The points are colour-coded by similarity to the chromosome assignment of *A. thaliana* gene models (grey=no match, black=chloroplast or mitochondria, light blue=chromosome 1, orange=chromosome 2, dark blue=chromosome 3, green=chromosome 4, and red=chromosome 5).

Most of the unigenes that could be mapped to the pseudomolecules with high sequence similarity hits (threshold value 1E-30) could be mapped to both A and C genomes, enabling a detailed analysis of the collinearity of the genomes. The positions in the respective genome of the 57,246 unigenes that could be mapped to both are illustrated in Fig. 1.

This shows the expected high degree of conservation of gene order, but there are numerous segmental rearrangements differentiating them. The extent of rearrangement is perhaps surprising given that they shared a common ancestor only 3.7 MYA [9], but this genome plasticity may be accounted for by their polyploid origins [10]. There is also a background of unigenes mapping to non-homoeologous positions in the A and C genomes, including two prominent segments shadowing each of the collinear blocks. To investigate the possible basis of these, 60 unigenes anchored to linkage group A1 were selected randomly and sequence similarity in the A and C genomes assessed by visual inspection of BLAST alignments. The results of this analysis (Additional file 8) showed that the apparent non-collinear mapping is most often an artefact caused by repetitive sequences, but sequences missing from one genome (i.e. copy number variation) are common, too. The latter explains the “shadow” collinear blocks as the best sequence match remaining after loss of a homoeologous sequence will be that of a paralogue; the hexaploid basis of each of the A and C genomes then results in two such paralogous segments being detected.
